# Ileocolonic-Targeted JAK Inhibitor: A Safer and More Effective Treatment for Inflammatory Bowel Disease

**DOI:** 10.3390/pharmaceutics14112385

**Published:** 2022-11-05

**Authors:** Vipul Yadav, Aileen House, Silvia Matiz, Laura E. McCoubrey, Kimberly A. Bettano, Leena Bhave, Meiyao Wang, Peter Fan, Siqun Zhou, Janice D. Woodhouse, Eirini Poimenidou, Liu Dou, Abdul W. Basit, Lily Y. Moy, Robert Saklatvala, Laxminarayan G. Hegde, Hongshi Yu

**Affiliations:** 1 Intract Pharma Ltd., London Bioscience Innovation Centre, 2 Royal College Street, London NW1 0NH, UK; 2 Merck & Co., Inc., 126 East Lincoln Avenue, P.O. Box 2000, Rahway, NJ 07065, USA; 3 UCL School of Pharmacy, University College London, 29-39 Brunswick Square, London WC1N 1AX, UK; 4 Karuna Therapeutics, Inc., 99 High St Floor 26, Boston, MA 02110, USA; 5 Treeline Biosciences, 500 Arsenal Street, Suite 201, Watertown, MA 02472, USA; 6 90TEN, Battersea Studios, 80 Silverthorne Road, London SW8 3HE, UK; 7 School of Pharmaceutical Sciences (Shenzhen), Sun Yat-Sen University, Guangzhou 510275, China; 8 Kallyope, 430 East 29th Street, 10th Floor, New York, NY 10016, USA

**Keywords:** JAK inhibitors, colonic drug delivery, drug stability, ulcerative colitis, Crohn’s disease, anti-inflammatory, targeting the large intestine, Phloral film coating technology

## Abstract

Janus kinase (JAK) inhibitors, such as tofacitinib (Xeljanz) and filgotinib (Jyseleca), have been approved for treatment of ulcerative colitis with several other JAK inhibitors in late-stage clinical trials for inflammatory bowel disease (IBD). Despite their impressive efficacy, the risk of adverse effects accompanying the use of JAK inhibitors has brought the entire class under scrutiny, leading to them receiving an FDA black box warning. In this study we investigated whether ileocolonic-targeted delivery of a pan-JAK inhibitor, tofacitinib, can lead to increased tissue exposure and reduced systemic exposure compared to untargeted formulations. The stability of tofacitinib in the presence of rat colonic microbiota was first confirmed. Next, in vivo computed tomography imaging was performed in rats to determine the transit time and disintegration site of ileocolonic-targeted capsules compared to gastric release capsules. Pharmacokinetic studies demonstrated that systemic drug exposure was significantly decreased, and colonic tissue exposure increased at 10 mg/kg tofacitinib dosed in ileocolonic-targeted capsules compared to gastric release capsules and an oral solution. Finally, in a rat model of LPS-induced colonic inflammation, targeted tofacitinib capsules significantly reduced concentrations of proinflammatory interleukin 6 in colonic tissue compared to a vehicle-treated control (*p* = 0.0408), unlike gastric release tofacitinib capsules and orally administered dexamethasone. Overall, these results support further development of ileocolonic-targeted tofacitinib, and potentially other specific JAK inhibitors in pre-clinical and clinical development, for the treatment of IBD.

## 1. Introduction

Inflammatory bowel disease (IBD) is a collective term used to describe two conditions: ulcerative colitis (UC) and Crohn’s disease (CD). Despite intense research focus over the last 100 years, there remains no cure for either UC or CD [1]. Furthermore, available IBD treatments are frequently ineffective in inducing or maintaining remission and can carry a high risk of adverse effects [2,3]. For instance, a systematic review found that 17% of UC patients require hospitalization within their first year of diagnosis, despite the vast majority being prescribed medication [4]. At 10 years after diagnosis, 66% UC patients were found to have required admission and around 14% required colectomy. Surgery rates are comparatively higher in CD than UC, with a meta-analysis estimating patients’ risk of surgery as 39.5% in the first 10 years of diagnosis [5]. Clearly, the need for surgery in IBD marks a failure for medications to adequately control disease. Both UC and CD can be chronic, relapsing, and progressive conditions; therefore, achieving effective treatment in the early stages of disease is crucial [3]. 

To address the shortcomings of traditional IBD drugs, such as the aminosalicylates, corticosteroids and immunosuppressants, several novel treatments have been developed in recent decades [3]. Monoclonal antibodies targeting tumour necrosis factor (TNF)-alpha, integrin, and interleukin (IL) 12–23 have provided important therapeutic options for moderate to severe IBD patients who fail to respond to first line therapy [6,7]. Biologics licensed for IBD have proven effective in inducing and maintaining disease remission; however, they are currently only available as parenteral formulations and are known to increase risk of immunogenicity, infections, and malignancy [8,9]. Emerging treatments for IBD include various biologics designed to improve upon current options (e.g., IL-2 and oral biologic formulations), and small molecule drugs, such as sphingosine 1 phosphate receptor modulators, phosphodiesterase 4 inhibitors, and Janus kinase (JAK) inhibitors [3]. 

Tofacitinib (Xeljanz^®^, Pfizer) and filgotinib (Jyseleca^®^, Galapagos) are currently the only JAK inhibitors approved for treatment of moderate to severe UC [10,11]. As a pan-JAK inhibitor, tofacitinib has affinity for the cytoplasmic JAK1, 2, and 3 enzymes, which are involved in the transduction of multiple pro-inflammatory cytokines related to IBD [12]. By binding to the JAK enzymes, tofacitinib prevents the phosphorylation of the JAK molecules and subsequently reduces transcription of pro-inflammatory cytokines. In clinical trials, tofacitinib has significantly outperformed placebo in the induction of UC remission and the maintenance of UC remission, by as much as 29.5%, proving itself an efficacious therapy. However, there are significant safety concerns surrounding the drug’s broad immunosuppressive activities [13]. Serious adverse events have been reported following long-term tofacitinib treatment, including reduction in leukocyte subset numbers, serious and/or opportunistic infections, increased low-density lipoprotein (LDL) cholesterol, and non-melanoma skin cancer [14,15,16]. Other safety trials have also reported cases of pulmonary embolism, increased risk of cardiovascular events, and death when compared with anti-TNF inhibitors [17,18]. This risk could be due to tofacitinib’s broad inhibition of multiple cytokines, which may provoke dysregulated cytokine signalling within important systemic processes [9]. For example, JAK2 blockade can impair immunoglobulin synthesis and T-cell development, thus increasing patients’ risk of infection. Further, inhibition of JAK1 may promote myocardial fibrosis, infarction, and malignant arrhythmia through activation of the interleukin 6 (IL-6) heart failure pathway [9,19]. 

Considering current evidence, delivering tofacitinib to the ileocolonic region could offer several benefits. Firstly, ileocolonic delivery of tofacitinib could reduce systemic drug exposure and thus limit adverse effects arising from systemic pan-JAK blockade [9]. The colonic epithelium can be more difficult for drugs to permeate than the small intestinal epithelium due to its double layer of mucus and lack of villi [20]. Secondly, ileocolonic delivery of tofacitinib could achieve higher drug concentrations at the disease site, potentially leading to superior treatment of UC [21]. A molecular design approach to ileocolonic-targeting was recently demonstrated with an azo pro-drug of tofacitinib, whereby the pro-drug was shown as an effective, and potentially safer, treatment for UC in a mouse model [22]. Formulation-guided ileocolonic drug delivery can be achieved by coating formulations with materials that remain intact during transit through the stomach and small intestine and selectively degrade in the distal GI tract, allowing site specific drug release [20,23]. Phloral^®^ is an example of such a coating, its pH and microbiota-sensitive composition have demonstrated consistent colonic targeting in human studies and have enabled its approval as a licensed product [24,25,26].

This study examined a Phloral^®^-coated tofacitinib formulation for the treatment of colonic inflammation. The stability of tofacitinib in the presence of cecal fluid was first measured to characterise any microbial drug metabolism [27]. Next, in vivo release characteristics of the ileocolonic-targeted formulation were compared to untargeted formulations, using imaging and pharmacokinetic (PK) profiling. The pharmacodynamic (PD) effects of the targeted vs. untargeted formulations were then compared in a lipopolysaccharide (LPS)-induced rat model of inflammation. This work aimed to explore whether the safety and efficacy of tofacitinib can be improved by formulating the drug for ileocolonic release.

## 2. Materials and Methods

### 2.1. Materials 

Tofacitinib citrate was synthesized at Merck & Co., Inc., Rahway, NJ, USA. Haemin, L-cysteine HCl, vitamin K, resazurin, boric acid, phosphate buffer saline, Nonidet P-40, dexamethasone, polysorbate 80, polysorbate 20, polyethylene glycol 400, fluorescamine, and sodium bicarbonate were obtained from Sigma Aldrich, Gillingham, UK. Sodium chloride and dipotassium hydrogen phosphate were obtained from Fisher Chemical, Loughborough, UK. Magnesium sulphate heptahydrate and calcium chloride hexahydrate were obtained from VWR, Poole, UK. Bile salts were from Fluka Analytical, Buchs, Switzerland. Barium sulfate was purchased from Alfa Aesar by Thermo Fisher Scientific, Haverhill, MA, USA. Methocel^®^ A4C premium was purchased from Dow Chemical Company, Midland, MI, USA. All other chemicals used were of high-performance liquid chromatography (HPLC)-grade and were used as received. 

### 2.2. Methods

#### 2.2.1. Animal Models

Healthy male Lewis rats were purchased from Charles River Laboratories (Wilmington, MA, USA), average weight 350 g (range 320–360 g). Animal studies were performed in accordance with the guidelines of the Institute for Laboratory Animal Research (ILAR). All studies were part of an institutional animal care and use committee (IACUC)-approved protocol and animals were housed in an AAALAC (American Association for Accreditation of Laboratory Animal Care) international accredited research facility and were performed in compliance with the Home Office standards under the Animals (Scientific Procedures) Act 1986, UK (P4AF0DB91). Animal welfare was prioritized during all activities involving their use, and the Animal Research: Reporting of In Vivo Experiments (ARRIVE) guidelines were consulted and observed. Rats were housed in groups at regulated temperature (25 °C) and relative humidity (50–60%) with regular light–dark cycles of 12 h. Animals were acclimatized for at least 7 days in the experimental unit before any studies commenced and were provided with unlimited access to food and water (except where overnight fasting was required before the morning of experiments, as in Section 2.2.7. 

Plasma was obtained from the inferior vena cava using a BD Tuberculin Syringe with a 25G Detachable Needle (BD309626) and placed in a BD Microtainer™ Capillary Blood Collector and BD Microgard™ Closure (BD 365974). Rats were euthanized via CO_2_ asphyxiation prior to the removal of intestinal fluids or tissue. Colonic tissue was excised starting at the cecum to the distal colon, flash frozen in liquid nitrogen, and stored at −80 °C prior to analysis.

#### 2.2.2. Ex Vivo Rats Colonic Microbiota Stability of Tofacitinib 

The susceptibility of tofacitinib to metabolism/bioaccumulation by microbiota was investigated to assess the drug’s stability in the colonic environment [28]. The cecum of healthy rats (male, *n* = 6) was obtained shortly following animal sacrifice and transferred to an anaerobic workstation (Electrotek 500TG workstation, Electrotek, West Yorkshire, UK) maintained at 37 °C and 70% relative humidity. The rats’ cecums were opened and fluid was collected using aseptic technique to preserve the fluid’s microbial composition. Next, the fluid was homogenised with basal media (ratio 1:4, composition as used by Hughes et al. [29] at 10,000 rpm and sieved through a mesh with 5 µm aperture. 

Tofacitinib was incubated at a concentration of 1 mg/mL in rat cecal slurry for 6 h within the anaerobic chamber (*n* = 3 reactions). Samples were taken at 0, 1, 2, 4, and 6 h and immediately quenched with ice-cold acetonitrile at a ratio of 1:3 to arrest microbial/enzyme activity. Quenched samples were then centrifuged (9600× *g*, 10 min, 4 °C) and the supernatant was collected for quantification of drug concentration using HPLC. The percentage remaining drug concentration at each timepoint was calculated by comparing timepoint concentrations to the concentration of tofacitinib added to the cecal slurries (1 mg/mL).

#### 2.2.3. HPLC Quantification of Tofacitinib

A 1260 Infinity II Series™ (Agilent Technologies, Cheadle, UK) HPLC system was used to measure the concentration of tofacitinib in rat cecal slurry. A mobile phase consisting of 0.1% phosphoric acid in water (A) and acetonitrile (B) was used via a gradient method, whereby %A: 95 and %B: 5 from 0.0–4.0 min; and %A: 80 and %B: 20 from 4.0–6.0 min. An Agilent Zorbax-Extend C18 column (50 × 4.6 mm, 1.8 μm) (Santa Clara, CA, USA) at 1.8 mL/min, with column temperature equilibrated at 40 ± 2 °C. 10 µL of tofacitinib samples were injected for each measurement and the drug was detected at 210 nm. Drug concentration was calculated by comparing the area under the curve (AUC) of the drug’s peak in samples to those produced with a calibration curve. 

#### 2.2.4. Formulation of Tofacitinib 

##### Ileocolonic-Targeted Capsules

Ileocolonic-targeted capsules were produced by coating Torpac size, 9h capsules with Phloral^®^. The Phloral^®^ technology utilises a continuous blend of Eudragit^®^ S and resistant starch (amylose and amylopectin), which together resist degradation in the upper GI tract and selectively breakdown in the human colon to achieve site-specific drug release [24,30]. In this mechanism, the dissolution of Eudragit^®^ S is triggered by the rise in pH between the small intestine and colon, and the digestion of resistant starch is achieved by the colonic microbiota. 

##### Liquid Formulation

Oral gavage formulations of tofacitinib were prepared by adding the drug directly to an appropriate volume of preprepared 0.5% methylcellulose/0.025% Polysorbate 20 in deionized water by weight (*w*/*w*). Sonication/magnetic stirring were performed as needed to achieve a uniform suspension or solution prior to oral administration to animals. 

#### 2.2.5. Investigation of Formulations’ Gastrointestinal Drug Release with Computed Tomography (CT) Imaging 

Male rats (*n* = 8) were fasted overnight before the day of CT imaging. Immediately prior to imaging, four rats were administered Phloral^®^-coated (ileocolonic-targeted) capsules and four were administered uncoated (untargeted) capsules via intra-gastric gavage, using a Torpac metal applicator (Fairfield, NJ, USA). All capsules (Torpac size, 9 h) were loaded with barium sulphate to facilitate their identification on CT images. Each rat was administered 500 µL water post-gavage to mimic the water usually taken with oral dosage forms and to encourage gastric emptying. The passage of capsules through the rats’ GI tracts was then measured by CT scanning the animals’ abdominal regions longitudinally, immediately, 3, 7, and 24 h after capsule administration. Animals’ access to food was recommenced after the 7-h imaging timepoint. CT scans were acquired with a GE eXplore Locus Ultra scanner (GE Appliances, Louisville, KY, USA) using a current of 50 mA (80 kVp for 16 s). Images were reconstructed using the SCANCO AxRecon 3.2.0.0. (Acceleware, Calgary, AB, Canada) with an image pixel size of 1200(x) × 400(y) × 1000(z) mm. The integrities of the coated and uncoated capsules through the GI tract were compared by a professional trained in radiography, allowing examination of the capsules’ point of dissolution.

#### 2.2.6. Pharmacokinetic Comparison of Targeted and Untargeted Formulations 

The delivery of tofacitinib to colonic tissue and plasma via coated capsules, uncoated capsules, and oral solution were compared in healthy rats (*n* = 6–12 per group). Rats were dosed with one of the three formulations at either 1 mg/kg or 10 mg/kg tofacitinib. 

A 10 mM stock solution of tofacitinib was made by dissolving a suitable quantity of the standard compound in dimethyl sulfoxide (DMSO). Standard working solutions at 1 and 0.05 mM were prepared by diluting suitable amounts of the stock solution (10 mM) with DMSO for the calibration standards (STD) and quality control (QC) samples preparation. Calibration standards and quality control samples were prepared by dispensing different volumes (ranging from 0.02 to 500 nL tofacitinib) of the standard working solutions using a HP D300e Digital Dispenser (Hewlett-Packard (HP), Palo Alto, CA, USA).

Rats were euthanized via CO_2_ asphyxiation prior to the removal of tissue. Pre-weighed colon tissue samples (average weight 1.50 g, range 1.20–1.68 g) were obtained in 24 well plates and 5 mL of artificial plasma was added to each well along with two metal beads. They were then homogenized using the Geno Grinder for 15 min. An amount of 50 µL of these tissue homogenates from dosed animals, or calibration standards and quality control samples in artificial plasma were prepared for analysis by protein precipitation. Artificial plasma was prepared by adding 4 g BSA in 100 mL 1X PBS. 

Plasma samples obtained from treated animals, calibration standards and quality control samples in blank plasma were prepared for analysis by protein precipitation. Protein precipitation was carried out by adding 200 μL of internal standard (IS) crashing solvent to 50 μL aliquots of individual samples. The IS solution was prepared by diluting 1 mL ampoule of Cerilliant IS MIX (diclofenac 200 µM, labetalol 200 µM, and imipramine 200 µM) in 1 L of acetonitrile. Samples were mixed by vortexing for 2 min and centrifuged at 3500 rpm for 5 min. The supernatant (200 μL) was transferred into a 96-well plate and injected into the LC-MS/MS for analysis of tofacitinib concentration. Chromatography was performed on a Waters Acquity HSS T3 (2.1 mm × 50mm, 1.8 µm) column at room temperature with an injection volume of 5 µL. The mobile phase consisting of a solvent A (0.1% formic acid in water) and solvent B (0.1% formic acid in acetonitrile) was delivered at a flow rate of 750 µL/min. The LC gradient started from 95/5 (A/B) and changed to 5/95 (A/B) from 0.25 to 1.75 min (ramp) and remaining constant to this ratio for 0.42 min (step). The gradient decreased to 95/5 (A/B) at 2.17 min (step) remaining constant to this ratio for 1.0 min. 

Detection of tofacitinib was carried out using a triple quadrupole tandem mass spectrometer (API 6500, Applied Biosystems) equipped with an electrospray interface (ESI). Ions were created in the positive ion mode setting the sprayer voltage at 5.0 kV and the ion source temperature at 500 °C. The common parameters and the nitrogen flow values for nebulizer gas (Gas 1), auxiliary gas (Gas 2), curtain gas and the gas for collision-activated dissociation (CAD) were set at 60, 60, 35, and 5, respectively. The Analyst 1.6.2 software (Applied Biosystems) was used to control the MS-MS system and MultiQuant 3.0.1 for data analyses. Detection of tofacitinib was performed in the multiple reaction-monitoring (MRM) mode and the following precursor to product ion pair *m*/*z* 313.5 → 173.1 (DP/CE: 70/50) was used for quantitation. The following MRM transitions were monitored for the IS, 329.200 → 162.100 (DP/CE: 76/37) 

Standard curve range for both plasma and artificial plasma standard curve was 0.0025 µM–10 µM and a weighting of 1/x2 was applied. 

#### 2.2.7. The Pharmacokinetics and Pharmacodynamic Effects of Tofacitinib Formulations in an Acute Rat Model of Inflammation

Male Lewis rats were fasted overnight before the day of experiment. Rats were chosen as an in vivo model in these experiments as they are commonly used for assessment of IBD investigatory treatments [31,32]. For the ascending dose oral solution study (results shown in Supplementary Material, Figures S1 and S2), at T0 rats were orally administered, by gavage, either 1 mg/kg, 3 mg/kg, or 10 mg/kg tofacitinib solution. To induce intestinal inflammation, rats were intraperitoneally injected with 1 mg/kg LPS at T1 h. Intraperitoneal injection of LPS is an accepted method for instigating inflammation in rats as pharmacodynamic endpoints [33]. At T4 h rats were sacrificed, and terminal plasma and intestinal tissue was extracted for analysis of tofacitinib concentration as per Section 2.2.6 Plasma and tissue IL-6 concentrations were quantified using a V-PLEX validated immunoassay kit (Meso Scale Discovery Proinflammatory Panel(rat) (K15059D), Meso Scale Diagnostics, Rockville, MD, USA). Controls used in this study were naïve rats (no induction of inflammation, placebo treatment (PBS)), treatment-free rats (LPS + vehicle (PBS)), and comparative treatment control rats (induction of inflammation, treatment with 1 mg/kg oral dexamethasone solution, formulated in PEG400/polysorbate 80 in deionized water (10/9/81). Rats not treated with LPS were instead injected with the same volume of PBS solution.

For the studies involving the tofacitinib capsules, at T0 rats (*n* = 8 per group) were orally administered, by gavage, one of the following tofacitinib formulations: a 1 mg/kg coated capsule, 3 mg/kg coated capsule, 10 mg/kg coated capsule, 10 mg/kg uncoated capsule, or 10 mg/kg solution. To induce intestinal inflammation, 1 mg/kg LPS was injected intraperitoneally into rats at T5 h, this timing was chosen based on the CT imaging results, to allow capsules to reach the intestines. Inflammation-free control rats were instead intraperitoneally injected with an equal volume of PBS. Controls used in this study were naïve rats (no induction of inflammation, vehicle treatment (PBS)), treatment-free rats (LPS + vehicle (PBS)), and comparative treatment control rats (induction of inflammation, treatment with 1 mg/kg oral dexamethasone solution, formulated in PEG400/polysorbate 80 in deionized water (10/9/81), at T4 h). Animal sacrifice occurred at T8 h (3 h after LPS administration). Terminal plasma and colonic tissue were subsequently excised, and levels of IL-6 quantified using the V-PLEX validated immunoassay kit.

#### 2.2.8. Data Analysis and Statistics 

Data produced during the experiments were stored, analysed, and plotted in GraphPad Prism (version 9.3.1, San Diego, CA, USA). A one sample *t*-test was used to determine if the stability of tofacitinib in the rat caecal slurry at the measured timepoints was significantly different to the starting drug concentration. To compare PK and PD results between formulation types, the means of each group were compared with a one-way ANOVA followed by Tukey’s multiple comparison tests. For all tests, a *p*-value of ≤0.05 was considered significant. In plots, the use of the * symbol confers: * *p* ≤ 0.05; ** *p* ≤ 0.01; *** *p* ≤ 0.001. Area under curves (AUCs) were calculated using GraphPad’s AUC tool and presented as total peak areas ± standard error. The maximum plasma concentration (C_max_) and the time at which maximum concentration occurred (T_max_) were obtained by inspection of the plasma concentration–time data. Other data in graphs and values quotes in text represent means ± standard deviation unless otherwise stated. 

## 3. Results

### 3.1. Tofacitinib Stability in Rat Cecal Slurry

**Figure 1:**
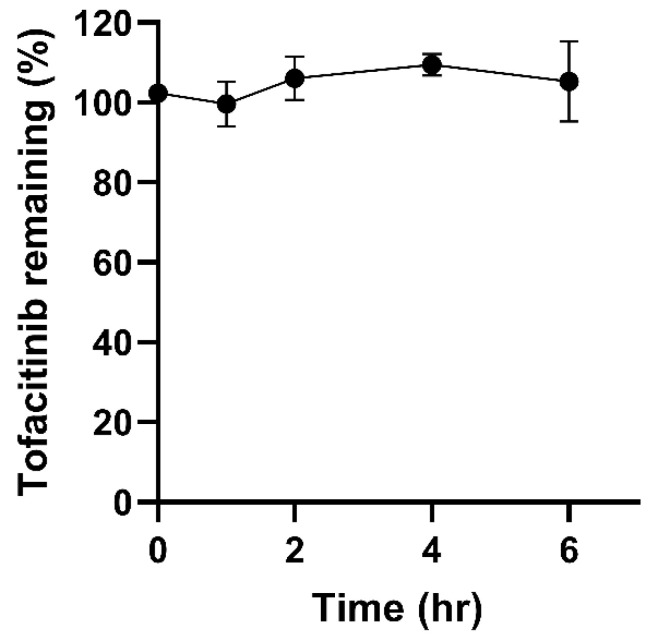
The stability of tofacitinib in rat caecal slurry over 6 h (*n* =3).

Figure 1 shows that tofacitinib was completely stable in the presence of rat colonic microbiota, with no significant differences noted between 100% stability and the remaining drug at any timepoint (*p* = 0.052). These results highlight that the drug would be stable for at least 6 h when delivered to the cecum/colon, and therefore was suitable for formulation in a ileocolonic-targeted capsule. 

Over 150 drugs are known to be chemically transformed by intestinal microbiota, thus it was important to examine tofacitinib’s susceptibility to microbial inactivation or toxification [34]. Tannergren et al. have demonstrated that drug stability in the presence of intestinal microbiota has strong correlation (R^2^ = 0.90) with the extent of colonic drug absorption [35]. As tofacitinib showed high microbial stability in this study, it has a strong chance of being absorbed into colonic tissue for local treatment of IBD. 

### 3.2. Comparison of Coated vs. Uncoated Capsules’ Integrity In Vivo 

**Figure 2:**
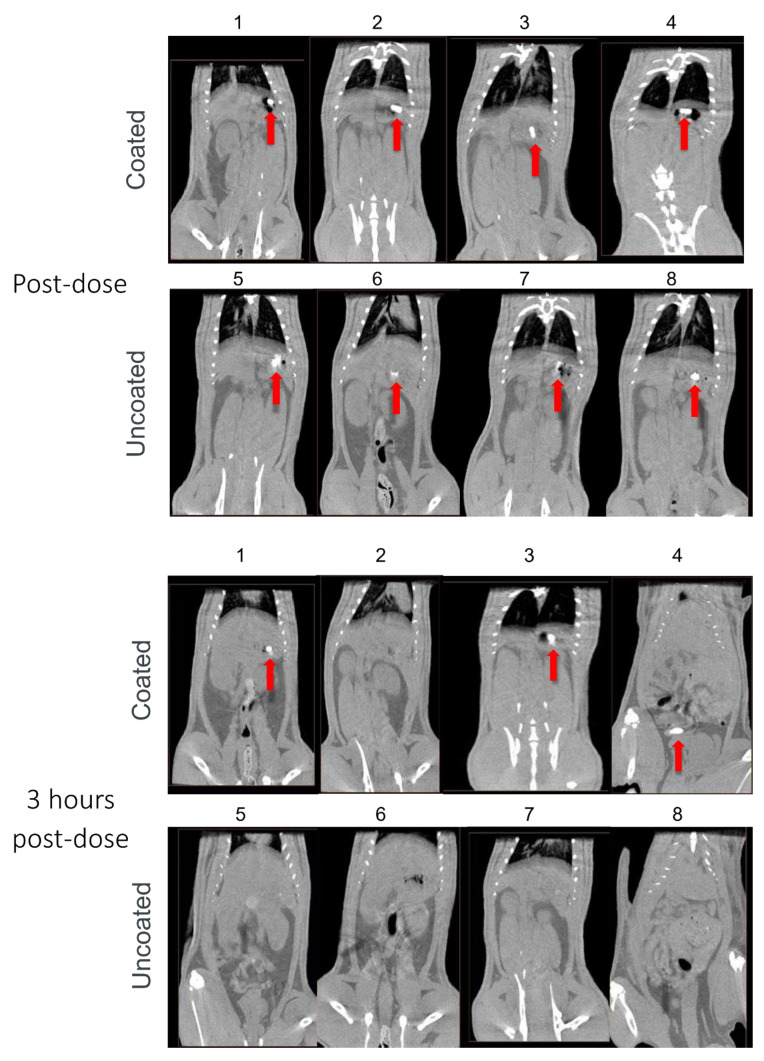

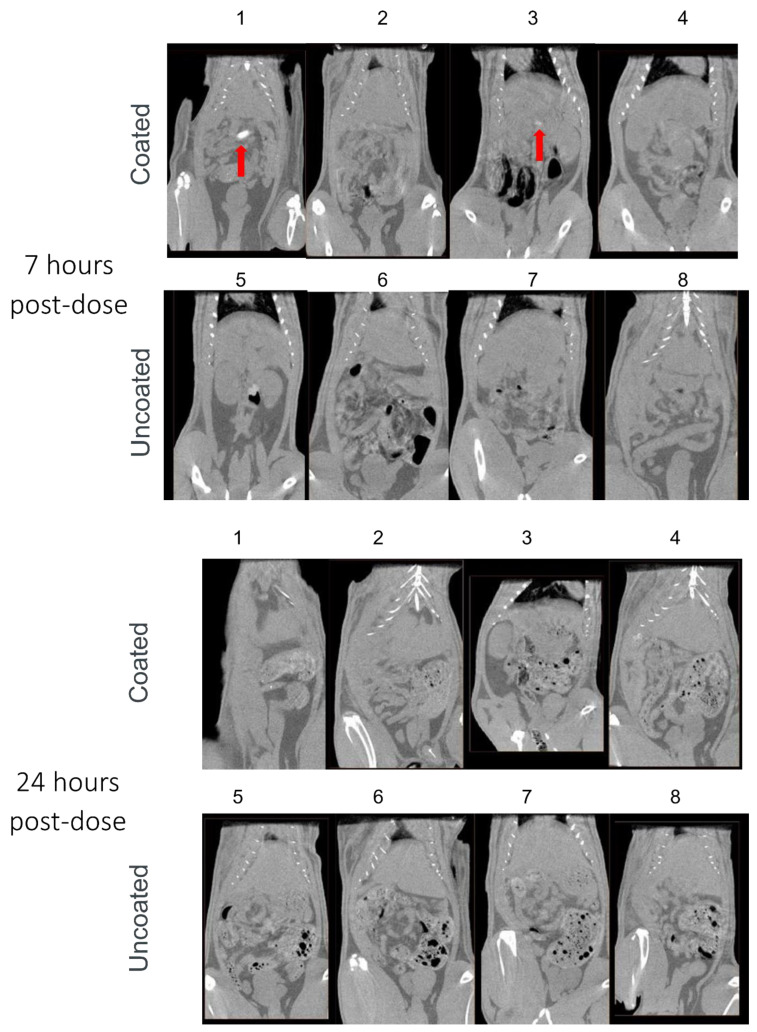
Computed tomography (CT) images captured immediately and 3, 7, and 24 h after administration of Phloral^®^-coated and uncoated capsules to rats (*n* = 4 each group; rats 1–4 received coated capsules, rats 5–8 received uncoated capsules). Contrast demonstrating the presence of intact/partially intact capsules in the stomach or intestines is indicated with red arrows.

We next evaluated the effect of Phloral^®^ coating on capsules using CT imaging to longitudinally track capsule transit through the GI tract. While the data represented is in 2D format, the investigator analysed each slice of the CT scan. Figure 2 shows how Phloral^®^-coated capsules resisted dissolution in the upper GI tract. Immediately after administration, all coated capsules were observed intact in the stomach (Figure 2: rats 1, 2, 3, 4). In comparison, at the same timepoint 3 of the 4 uncoated capsules appeared to have begun disintegration (rats 5, 6 and 7, Figure 2), demonstrated by diffuse contrast in their CT images. At 3 h post-dose, all uncoated capsules had dissolved as no defined contrast was visible at any point in the GI tract. In juxtaposition, 3 out of 4 of the coated capsules remained intact, with one capsule observed to have dissolved in the stomach of rat 2. At 3 h, two of the coated capsules remained intact in the stomach (rats 1 and 3), and one capsule was detected in the mid-distal small intestinal (rat 4). At 7 h after administration, only the coated capsule in rat 1 remained fully intact (observed in the small intestine) and coated capsules had dissolved in rat 4. The coated capsule in rat 3 was judged as partially dissolved at 7 h, due to defined but slightly less complete contrast visible in the intestines. At the final timepoint of 24 h, all coated and uncoated capsules were verified to have dissolved. Contrast visible in the intestines at 24 h could represent remaining contrast from the dissolved capsules, or food, which was reintroduced after the 7-h timepoint.

The results from the CT imaging analyses demonstrated that coated capsules remained intact in the upper GI tract for significantly longer and dissolved in the distal intestine compared to uncoated capsules that dissolved immediately in the stomach. All uncoated capsules were seen to degrade in the stomach of the rats before 3 h, with none visualized as intact in the intestines at any timepoint (Figure 2). In juxtaposition, 3/4 of the coated capsules were visualised intact in the intestines, indicating that they had successfully resisted degradation in the stomach and proximal small intestine. CT assessment of capsule transit through the GI tract correlated well to a pilot study conducted with capsules filled with methylene blue dye, where dye localization was monitored terminally (data not shown). The Phloral^®^ technology is designed to deliver drugs to the human colon [24]. Phloral^®^ has been shown to resist degradation in the proximal gut and selectively release drug in the colon, where it encounters the dual triggers of luminal pH > 7.0 and polysaccharide-metabolizing microbiota [20]. Rats naturally present a higher pH profile in the early small intestine (>7) as well as higher densities of microbiota in the stomach and small intestine compared to humans. This may explain why the coated capsules mostly degraded in the small intestine in this study despite the highest coating thickness applied [36,37]. In humans, Phloral^®^ has been shown to reliably deliver drugs specifically to the colon in both fed and fasted states [38]. 

### 3.3. Pharmacokinetic Comparison of Targeted and Untargeted Formulations in Healthy Rats

#### 3.3.1. Systemic Drug Exposure

**Figure 3:**
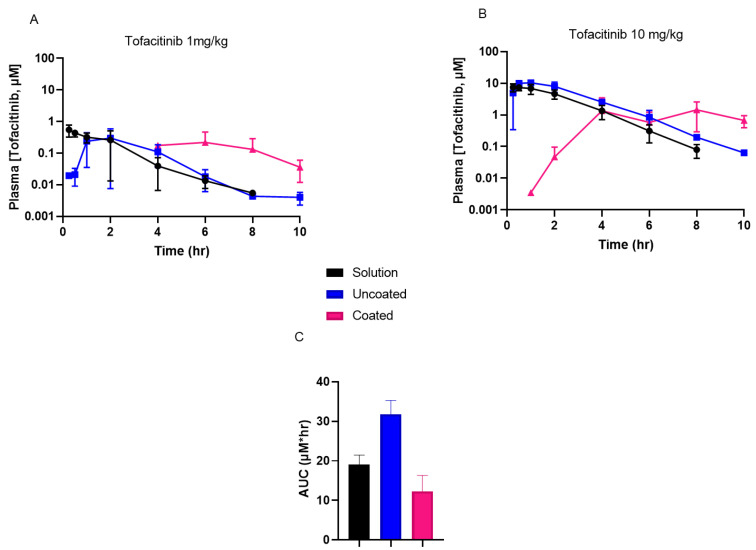
(**A**,**B**): Tofacitinib plasma concentrations recorded in rats following oral administration of a solution, uncoated capsules, and coated capsules dosed at either 1 mg/kg (**A**) or 10 mg/kg (**B**) tofacitinib. (**C**): Area under the curves (AUCs) of the 10 mg/kg tofacitinib formulations’ plasma concentration–time results (from data shown in (**B**)). AUC solution vs. uncoated *p* ≤ 0.0269 and coated vs. uncoated *p* ≤ 0.0011. C_max_ solution vs. coated *p* ≤ 0.0210 and uncoated vs. coated *p* ≤ 0.019.

To better understand the PK properties associated with Phloral^®^-coated capsules compared to uncoated capsules or the solution formulation, tofacitinib plasma concentrations were determined following administration (Figure 3A,B, tabulated data in Table S1). Coated capsules achieved delayed maximal plasma concentrations (T_max_) compared to the uncoated capsules and solution for both 1 mg/kg and 10 mg/kg doses. For the 10 mg/kg dose, the maximal tofacitinib plasma concentration (C_max_) for coated capsules was significantly lower than for the solution and uncoated capsules (*p* < 0.0001). In addition, the AUC of the plasma concentration–time curve for the coated capsules (AUC: 12.3 ± 4.0) was numerically lower than for the solution (AUC: 19.1 ± 2.4) and significantly lower than for the uncoated capsules (AUC: 31.8 ± 3.5, *p* ≤ 0.0011) (Figure 3C). For the 1 mg/kg dose, plasma measurements for the coated capsules were recorded from the 4-h timepoint due to earlier timepoint concentrations being below the bioanalytical quantification limit. Therefore, the AUC for animals receiving the 1 mg/kg coated capsules cannot be compared with the solution and uncoated capsules, as collection of samples from the latter formulations included additional earlier timepoints. However, the results for the 10 mg/kg dose show that administration of coated capsules resulted in lower systemic drug exposure compared to the untargeted formulations. This is important, as reduced systemic drug exposure could lead to fewer adverse effects and thus improve the safety profile of untargeted tofacitinib formulations. 

#### 3.3.2. Colonic Tissue Exposure 

**Figure 4:**
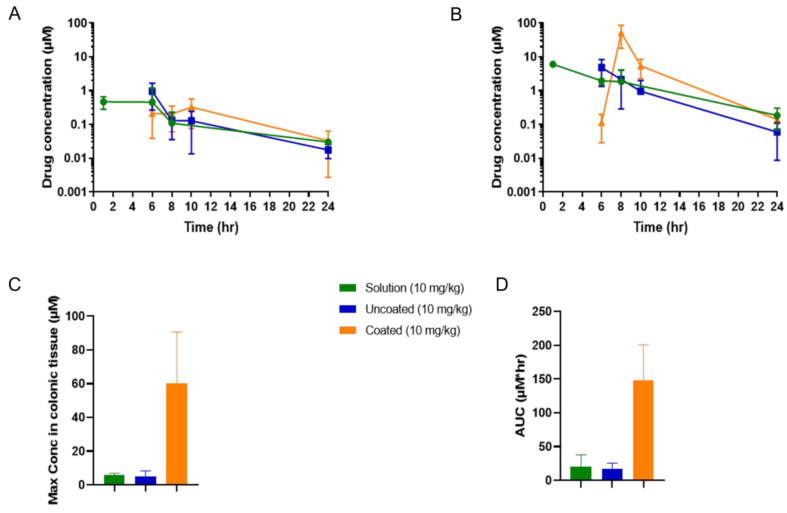
Concentrations of tofacitinib in rats’ colonic tissue following administration of a solution, uncoated capsules, and coated capsules dosed at either 1 mg/kg (**A**) or 10 mg/kg (**B**) tofacitinib. (**C**): The maximal tofacitinib concentrations of the 10 mg/kg solution, uncoated capsules, and coated capsules measured in colonic tissue. (**D**): Area under the curves (AUCs) of the 10 mg/kg tofacitinib formulations’ colonic tissue concentration–time results (from data shown in (**B**), measurement starting at 6 h post-dose). AUC solution vs. coated *p* ≤ 0.0350 and uncoated vs. coated *p* ≤ 0.0312. Maximal concentration solution vs. coated *p* ≤ 0.0210 and uncoated vs. coated *p* ≤ 0.019.

The overall colonic tissue concentrations of tofacitinib were similar following administration of the three formulations when dosed at 1 mg/kg (Figure 4A, tabulated data shown in Table S2). However, the Phloral^®^-coated capsules attained a significantly higher tofacitinib peak concentration in colonic tissue compared to the untargeted (uncoated and solution) formulations when dosed at 10 mg/kg, respectively (*p* = 0.021 for solution, *p* = 0.019 for uncoated capsules, Figure 4B,C). This demonstrates that at the higher dose the coated capsules achieved higher local drug concentrations, an attribute that could be leveraged to enhance local drug action and limit systemic exposure in IBD. Indeed, administration of the 10 mg/kg coated capsules resulted in significantly higher maximal tofacitinib concentrations in colonic tissue than plasma (*p* = 0.0001). The maximal concentration achieved from the 10 mg/kg administration in coated capsules occurred at 8 h, suggesting that most tofacitinib release occurred at this time. In comparison, the maximal concentrations following administration of the 10 mg/kg solution and uncoated capsules was earlier (1–6 h), indicating that the tofacitinib in these formulations reached the colon more quickly. This could be due to the solution and uncoated capsules releasing earlier in the GI tract, leading to the drug being absorbed into systemic circulation and distributing into colonic tissue from plasma. The 10 mg/kg coated capsules resulted in the highest overall tofacitinib exposure to colonic tissue, as demonstrated by a higher AUC measured from 6 h (AUC coated capsules: 148.0, solution 19.9, uncoated capsules 17.2, Figure 4D). 

### 3.4. The Pharmacokinetics and Pharmacodynamics of Tofacitinib Formulations in an Acute Rat Model of Inflammation

#### 3.4.1. Targeted Capsule Dose Response Study

**Figure 5:**
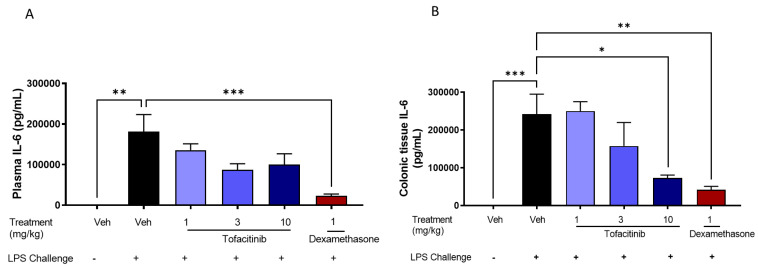
Plasma (**A**) and colonic tissue (**B**) levels of interleukin 6 (IL-6) in rats with LPS-induced inflammation 8 h after administration of Phloral^®^-coated tofacitinib capsules at 1, 3, and 10 mg/kg (3 h after LPS treatment). Controls included naïve rats (no LPS, treatment with phosphate buffered saline (PBS)); LPS + vehicle (LPS administration, treatment with PBS); and LPS + 1 mg/kg dexamethasone solution. *n* = 8 per treatment group.

Based on the PK profile, we designed a PKPD study to evaluate the effect of tofacitinib in coated capsules on plasma and colonic tissue PK, as well as LPS-induced plasma and colonic IL-6 levels (PD). Figure 5 shows the effect of increasing tofacitinib dose within Phloral^®^-coated capsules on the concentrations of IL-6 in rat plasma and colonic tissue. In plasma, there was no significant decrease in IL-6 levels with increasing tofacitinib dose. In colonic tissue, the 10 mg/kg coated tofacitinib capsules resulted in significantly lower IL-6 levels than the 1 mg/kg coated tofacitinib capsules (*p* = 0.0012) and the vehicle (*p* = 0.0123). These findings reflect the ability of the Phloral^®^ technology to selectively deliver sufficient tofacitinib dose to the colon, as the effect of drug dose on IL-6 was only apparent in colonic tissue. The 10 mg/kg tofacitinib capsules also significantly lowered colonic IL-6 levels compared to the untreated control (*p* = 0.012), to a statistically similar extent as dexamethasone. Unlike tofacitinib, dexamethasone also lowered plasma IL-6 compared to the untreated control, thus showing that its action was not selective to the colon. These results highlight that ileocolonic-targeted tofacitinib can reduce inflammatory markers in the colon, whilst exerting insignificant effects in systemic circulation. Based on these findings, the 10 mg/kg coated tofacitinib capsules were selected for comparison with untargeted oral formulations. 

The effect of increasing tofacitinib dose within oral solutions is presented in the Supplementary Materials (Figures S1 and S2). There was significant decrease in plasma and colonic tissue IL-6 levels with increasing tofacitinib dose, except that the decrease in IL-6 levels was maximal at 1 mg/kg in colonic tissue with no differences between the 1 and 10 mg/kg doses. 

#### 3.4.2. Comparison of Targeted vs. Untargeted Formulations 

**Figure 6:**
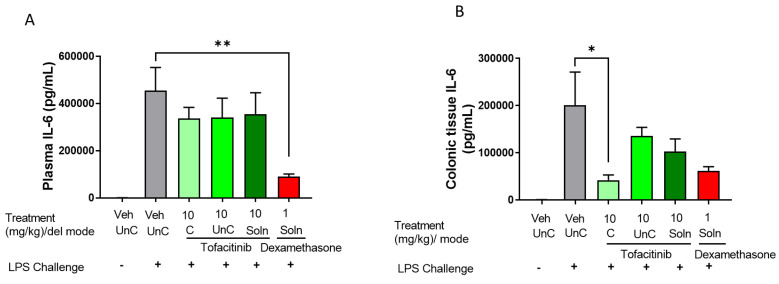
Plasma (**A**) and colonic tissue (**B**) concentrations of interleukin 6 (IL-6) in rats with LPS-induced inflammation 8 h after administration of ileocolonic-targeted (coated capsule, C), and untargeted (uncoated capsule, UnC) tofacitinib capsules or 4 h after tofacitinib solution (Soln). Controls included naïve rats (no LPS, treatment with phosphate buffered saline (PBS)); LPS + vehicle (LPS administration, treatment with PBS); and LPS + 1 mg/kg dexamethasone solution. *n* = 8 for each treatment group.

In an effort to compare the effect of targeted vs. untargeted formulations on systemic and colonic inflammatory markers, IL-6 levels were assessed in rat plasma and colonic tissue (Figure 6). Phloral^®^-coated tofacitinib capsules had no significant effect on IL-6 in plasma at the 10 mg/kg dose, though did significantly lower colonic tissue IL-6 compared to the untreated control (*p* = 0.0408). This reduction was not observed following administration of the uncoated tofacitinib capsules or tofacitinib solution at the 8 h analysis. Dexamethasone solution was the only formulation to significantly reduce plasma IL-6 levels compared to the untreated control after 8 h (*p* = 0.0072). Despite this, the dexamethasone solution did not significantly alter IL-6 concentration in colonic tissue at the 8 h analysis. It is worth noting that dexamethasone and 10 mg/kg tofacitinib solution did lower plasma and IL-6 levels in the oral solution experiment (Figure S2), where IL-6 levels were assessed 4 h post-dose. This highlights that the corticosteroid and tofacitinib solution may rapidly reduce both local and systemic IL-6. These results suggest that ileocolonic-targeted tofacitinib could preferentially control colonic inflammation without affecting systemic circulation following a single drug dose. As such, development of ileocolonic-targeted tofacitinib may be a promising method for the reduction of systemic side effects in IBD treatment, as therapeutic drug concentrations can be optimised in colonic tissue whilst limiting systemic exposure. Indeed, a recent study found that colonic tissue exposure to tofacitinib correlated with 30 UC patients’ remission status at week 16, which was not observed for plasma exposure [39]. These findings highlight that optimising concentration of JAK inhibitors within the colon could be a key determinant of therapeutic success in IBD. Further, intra-cecal delivery of tofacitinib in rats has been shown to enable the reduction of drug dose due to optimised colonic concentrations [40]. Targeting tofacitinib to the colon could reduce the incidence of the off-target adverse effects currently associated with the drug [17]. To further explore the colon-preferential action of Phloral^®^-coated tofacitinib capsules, it would be beneficial to test the effect of the formulation on more markers of inflammation as well as measurements of off-target adverse effects. 

## 4. Conclusions

This study outlines the development and PK/PD evaluation of a targeted oral delivery approach of the pan-JAK inhibitor tofacitinib for release in the ileocolonic region, the site of inflammation in UC. Using Phloral^®^, a clinically validated colon-targeting coating that can be seamlessly prepared and applied to solid oral dosage forms, we demonstrated that distal intestinal delivery of tofacitinib capsules to rats led to superior colonic tissue drug concentrations and reduced systemic exposure, compared to gastric release formulations, which represent the currently approved form of tofacitinib. Higher colonic tissue concentrations of tofacitinib translated into significant reductions in colonic tissue IL-6 levels in an LPS-induced model of inflammation, without impacting systemic IL-6 levels. Such a PK profile could have a marked impact on improving the safety profile of JAK inhibitors as a class whilst maintaining or enhancing their therapeutic efficacy in the treatment of GI inflammation. Moreover, Phloral^®^ is a simple and robust formulation composed of GRAS ingredients that eliminates the need for further preclinical toxicity studies of tofacitinib, allowing rapid scale up for testing clinical safety and efficacy and improving patient access. To this end, additional preclinical studies to evaluate the efficacy of other more specific JAK inhibitors like JAK-1 and TYK-2 using targeted delivery should be performed in the future, including large animal PK studies. Outcomes of such studies could potentially facilitate the realisation of safer and more effective oral JAK inhibitor formulations that can improve patient care in chronic GI inflammatory diseases. 

## Data Availability

Not applicable.

## Supplementary Materials

The following supporting information can be downloaded at: https://www.mdpi.com/article/10.3390/pharmaceutics14112385/s1, Figure S1: Plasma (A) and colonic tissue (B) tofacitinib concentrations following administration of 1 mg/kg, 3 mg/kg, and 10 mg/kg solutions to rats with acute intestinal inflammation. Dashed lines represent the lower limits of tofacitinib quantification (top) and detection (IC50 = 39 nM, PBMC, IL-7Stim (high serum). C: Area under the curves (AUCs) of the tofacitinib solutions’ plasma concentration–time results (from data shown in S1A). Figure S2: Plasma (A) and colonic tissue (B) concentrations of interleukin 6 (IL-6) in rats with LPS-induced inflammation 4 h after administration of oral solutions loaded with varying strengths of tofacitinib or dexamethasone, the latter acting as a therapeutic control. Naïve rats were LPS-free and treated with phosphate buffered saline (PBS). LPS + vehicle rats were administered PBS as a treatment, thereby acting as an untreated control. Table S1: Tofacitinib plasma concentrations recorded in rats following oral administration of a solution, uncoated capsules, and coated capsules dosed at either 1 mg/kg or 10 mg/kg tofacitinib. Results shown in Figure 3 in graphical form. Table S2: Tofacitinib colon concentrations recorded in rats following oral administration of a solution, uncoated capsules, and coated capsules dosed at either 1 mg/kg or 10 mg/kg tofacitinib. Results shown in Figure 4 in graphical form. Ref [41] has been cited in the Supplementary Materials.
