# Supplementary material for: Ileocolonic-Targeted JAK Inhibitor: A Safer and More Effective Treatment for Inflammatory Bowel Disease
Source: Pharmaceutics. 2022 Nov 5;14(11):2385. doi: 10.3390/pharmaceutics14112385 (PMC9698010; doi:10.3390/pharmaceutics14112385)
Supplement: Supplementary file 1 [file pharmaceutics-14-02385-s001.zip › pharmaceutics-1903638-supplementary.pdf]

Supplementary material

Figures S1A and S1B display the plasma and colonic tissue concentrations of tofacitinib following administration of three tofacitinib solutions of varying strength. Firstly, it is apparent that the plasma and colon tofacitinib concentrations in the acute inflammation model were similar to that observed in healthy rats (Figures 3 and 4). It is also clear that formulation strength affected plasma drug concentrations in the inflammation model. At 1-hour post-dose, the 10 mg/kg solution resulted in plasma tofacitinib concentrations significantly higher than the 1 mg/kg and 3 mg/kg solutions ($p < 0.0001$). However, the difference between 1 mg/kg and 3 mg/kg doses were statistically insignificant ($p = 0.219$). These relationships were reflected again at 4-hours post-dose, with the 10 mg/kg leading to higher plasma concentrations than the 1 mg/kg and 3 mg/kg solutions ($p = 0.0004$ and 0.0068, respectively). At 8-hours post-dose, only the difference between the 1 mg/kg and 10 mg/kg doses were significantly different ($p = 0.004$). The AUCs of the plasma drug concentrations over the 8-hour study period reflect the differences in total plasma drug exposure (Figure S1C). In comparison, tofacitinib concentrations in colonic tissue were less affected by the formulation strengths. At 1-hour post-dose, the colonic tissue tofacitinib concentrations following the 3 mg/kg and 10 mg/kg formulations were significantly higher than the 1 mg/kg formulation ($p = 0.034$ and 0.029, respectively). However, at all subsequent timepoints colonic drug concentrations were statistically indifferent. It is likely that plasma drug concentrations were more sensitive to alterations in formulation strength because the formulations were solutions. As such, a significant proportion of tofacitinib was likely absorbed into plasma before reaching colonic tissue, as oral solutions are not designed to deliver drugs specifically to the colon. As displayed in Figure 4B, targeted capsules resulted in higher colonic drug concentrations than oral solution. Even when administered as a solution, these data demonstrate that tofacitinib dose should be chosen with the desired plasma and tissue concentrations in mind. Indeed, the 3 mg/kg and 10 mg/kg solutions facilitated plasma and colonic tissue concentrations above the lower limit of quantification (0.0025 µm) for 8- and 24-hours post-dose, respectively.

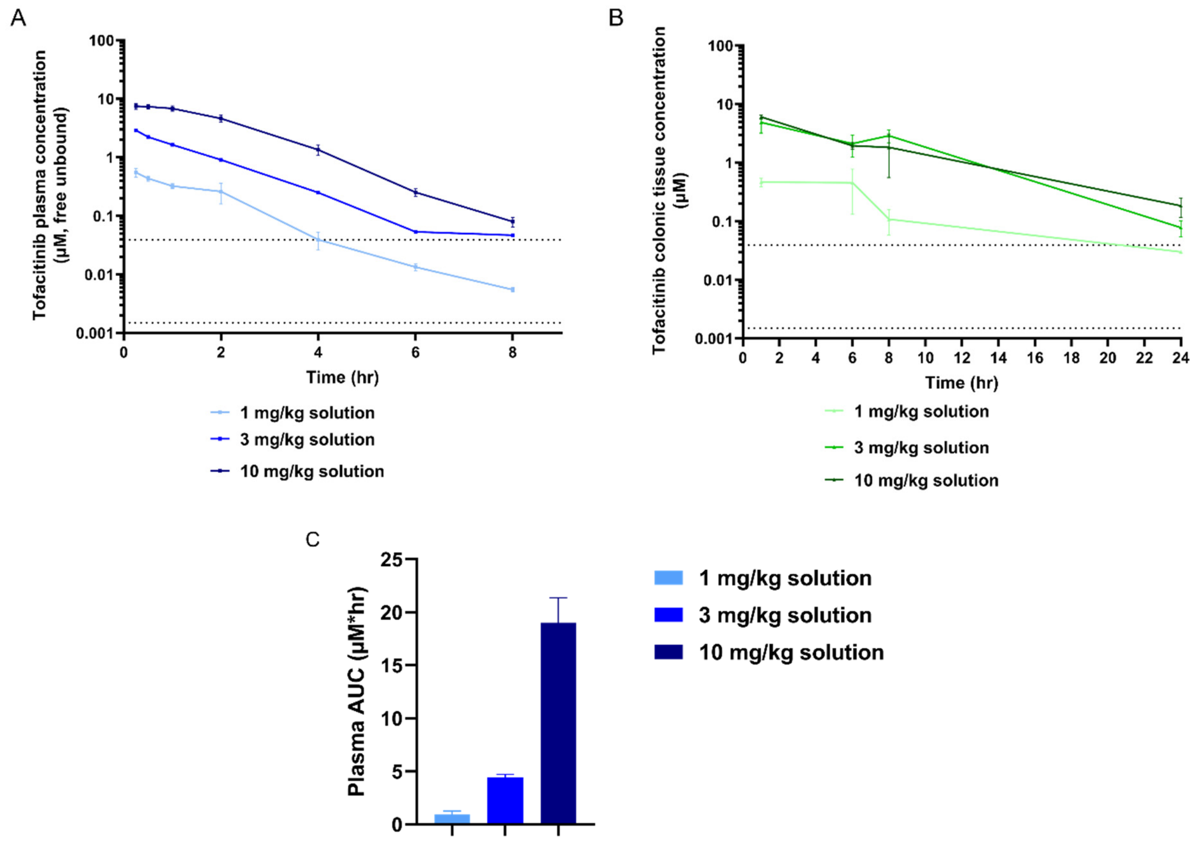

Figure S1. Plasma (A) and colonic tissue (B) tofacitinib concentrations following administration of 1 mg/kg, 3 mg/kg, and 10 mg/kg solutions to rats with acute intestinal inflammation. Dashed lines represent the lower limits of tofacitinib quantification (top) and detection (IC50=39 nM, PBMC, IL-7Stim (high serum). (C): Area under the curves (AUCs) of the tofacitinib solutions' plasma concentration-time results (from data shown in S1A).

Figure S2 shows the concentrations of IL-6 in rats' plasma and colonic tissue 4 hours after administration of varying doses of tofacitinib solution, a PBS control, or 1 mg/kg dexamethasone. IL-6 is a proinflammatory cytokine, therefore samples with lower systemic and intestinal IL-6 concentrations represent rats with superiorly controlled inflammation [1]. IL-6 levels in plasma (Figure S2A) were significantly lower following administration of 1 mg/kg dexamethasone solution, compared to the LPS + vehicle control ($p < 0.0001$). This was also observed for the tofacitinib solutions, as all three doses had statistically lower IL-6 plasma levels compared to the treatment-free control ($p = 0.024$ for 0.1 mg/kg; $p = 0.003$ for 1 mg/kg; $p = 0.0001$ for 10 mg/kg). Interestingly, there was no significant difference between plasma IL-6 levels in rats administered dexamethasone compared to the tofacitinib solutions. This demonstrates that tofacitinib effectively lowered systemic IL-6 to a similar degree as the established corticosteroid. In plasma, there was no significant difference between doses of tofacitinib. In colonic tissue dose-response relationships were more apparent, as the 1 and 10 mg/kg tofacitinib doses resulted in significantly lower IL-6 than the 0.1

mg/kg tofacitinib ($p = 0.046$ and 0.040, respectively, Figure S2B). There was no statistical difference between the 1 and 10 mg/kg tofacitinib doses, highlighting that in solution form, there was no therapeutic benefit of increasing tofacitinib dose > 1 mg/kg in this experiment. Both the 1 and 10 mg/kg tofacitinib solutions significantly lowered colonic tissue IL-6 compared to the treatment-free control ($p = 0.0001$ in both cases), as did dexamethasone ($p = 0.0002$). Favourably, there was no significant difference between the colonic IL-6 concentrations following administration of 1 and 10 mg/kg tofacitinib compared to the naïve rats, which had no induction of LPS inflammation. This demonstrates that, even in an untargeted solution, tofacitinib is effective at reducing an inflammatory marker in colonic tissue.

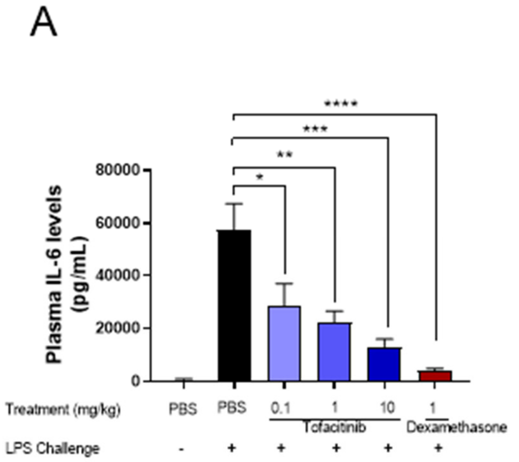

Figure S2. Plasma (A) and colonic tissue (B) concentrations of interleukin 6 (IL-6) in rats with LPS-induced inflammation 4 hours after administration of oral solutions loaded with varying strengths of tofacitinib or dexamethasone, the latter acting as a therapeutic control. Naïve rats were LPS-free and treated with phosphate buffered saline (PBS). LPS + vehicle rats were administered PBS as a treatment, thereby acting as an untreated control.

Table S1. Tofacitinib plasma concentrations recorded in rats following oral administration of a solution, uncoated capsules, and coated capsules dosed at either 1 mg/kg or 10 mg/kg tofacitinib. Results shown in Figure 3 (values shown as mean ± SEM).

Plasma Pharmacokinetics of Tofacitinib in male Lewis rats						
	Coated		Un-coated		Solution	
mg/kg (n)	1 (6)	10 (12)	1 (6)	10 (12)	1 (12)	10 (12)
$AUC_{(0-x)}$ (µM*Hours)	0.91± 0.42	12.32 ± 4.03	0.91 ± 0.36	31.78 ± 3.47	0.98 ± 0.29	19.12 ± 2.37
Maximum conc. (µM)	0.22 ± 0.30	1.44 ± 0.23	0.30 ± 0.04	10.38 ± 1.45	0.54 ± 0.08	77.36 ± 1.27
T_{max} (h)	6.33	5.30	1.20	0.83	0.63	0.42

Table S2. Tofacitinib colon concentrations recorded in rats following oral administration of a solution, uncoated capsules, and coated capsules dosed at either 1 mg/kg or 10 mg/kg tofacitinib. Results shown in Figure 4 (values shown as mean ± SEM).

Colon Pharmacokinetics of Tofacitinib in male Lewis rats						
	Coated		Un-coated		Solution	
mg/kg (n)	1 (6)	10 (6)	1 (6)	10 (6)	1 (6)	10 (6)
$AUC_{(0-x)}$ (µM*Hours)	3.43 ± 1.78	148.0 ± 52.76	2.37 ± 1.07	17.15 ± 8.51	3.98 ± 2.24	39.85 ± 18.01
Maximum conc. (µM)	0.32 ± 0.06	60.24 ± 13.54	0.96 ± 0.22	4.79 ± 1.03	0.47 ± 0.11	6.03 ± 1.25
T_{max} (h)	8	8	6	6	1	1

1. Mavropoulou, E. et al. (2020) Association of serum interleukin-6 and soluble interleukin-2-receptor levels with disease activity status in patients with inflammatory bowel disease: A prospective observational study. PLOS ONE 15 (5), e0233811.
